# Black Tea High-Molecular-Weight Polyphenol Stimulates Exercise Training-Induced Improvement of Endurance Capacity in Mouse via the Link between AMPK and GLUT4

**DOI:** 10.1371/journal.pone.0069480

**Published:** 2013-07-26

**Authors:** Tomoaki Eguchi, Chiaki Kumagai, Takashi Fujihara, Thoru Takemasa, Tetsuo Ozawa, Osamu Numata

**Affiliations:** 1 Graduate School of Life and Environmental Sciences, University of Tsukuba, Tsukuba, Japan; 2 Graduate School of Comprehensive Human Sciences, University of Tsukuba, Tsukuba, Japan; 3 The Shizuoka Organization for Creation of Industries, Shizuoka, Japan; University of Texas Health Science Center at San Antonio, United States of America

## Abstract

Aerobic exercise can promote “fast-to-slow transition” in skeletal muscles, i.e. an increase in oxidative fibers, mitochondria, and myoglobin and improvement in glucose and lipid metabolism. Here, we found that mice administered Mitochondria Activation Factor (MAF) combined with exercise training could run longer distances and for a longer time compared with the exercise only group; MAF is a high-molecular-weight polyphenol purified from black tea. Furthermore, MAF intake combined with exercise training increased phosphorylation of AMPK and mRNA level of glucose transporter 4 (GLUT4). Thus, our data demonstrate for the first time that MAF activates exercise training-induced intracellular signaling pathways that involve AMPK, and improves endurance capacity.

## Introduction

Tea is the most popular beverage in the world. Recent studies show that tea components, such as caffeine and polyphenols, have various beneficial effects at the physiological and cellular levels, in particular, anti-oxidation [1], anti-obesity [2], and anti-aging [3] effects. Fermented tea contains novel, high-molecular-weight polyphenol referred to as mitochondria activation factors (MAF), which can activate mitochondrial respiration, at least in a ciliated protozoan *Tetrahymena pyriformis* [4]. The number-range and the weight-range molecular weights of MAF are 9–18 kDa and 15–25 kDa, respectively [4]. Structural analysis showed that MAF is a heterogeneous polymer of flavan-3-ols and flavan-3-ol gallates with intermonomeric linkages of B-ring to B-ring and C-ring to A-ring [4].

A recent study revealed that MAF activates mitochondrial respiration (Fig. S1), and increases expression of PGC-1α, PPARδ, and mitochondrial proteins in C2C12 myotubes (Figs. S2 and S3). Therefore, MAF might be useful to induce fast-to-slow transition, to increase aerobic metabolism capacity in skeletal muscle and to improve endurance capacity. Skeletal muscles are composed of slow fibers (type I), which can continually contract for hours, and fast fibers (type II), which have higher contractile velocity but lower resistance to fatigue. Type II fibers are further subdivided into 3 groups (IIA, IIB and IID/X) based on the predominant myosin heavy chain (MHC) isoform each contains (MHC IIa, IIb and IId/x, respectively) [5]. IIA and IIB fibers have high and low oxidative metabolism capacity, respectively, whereas IID/X fibers are an intermediate type [5].

Oxidative skeletal muscles, which contain type I and type IIA fibers, are characterized by high density and activity of mitochondria, high amounts of myoglobin, active glucose uptake and fatty acid β-oxidation, and high capillary density. In contrast, muscles rich in IIB fibers have high anaerobic glycolytic capacity but low aerobic metabolism [6]. Continuous aerobic exercise can increase the proportion of oxidative fibers, a process known as “fast-to-slow transition”.

AMPK is a key factor in the fast-to-slow transition [7], [8]. It is a heterotrimeric serine-threonine kinase, which consists of the catalytic α subunit (α1, α2), scaffolding β subunit (β1, β2) and nucleotide-binding γ subunit (γ1, γ2, γ3) [9], [10]. AMPK senses the AMP/ATP ratio *via* AMP binding to its γ subunit; AMP binding induces α subunit phosphorylation on Thr172 by AMPK kinases. Thr172 phosphorylation activates AMPK [11], [12]. Mice lacking both AMPK β isoforms in skeletal muscles have drastically reduced exercise capacity, muscle mitochondria content and contraction-stimulated glucose uptake [13]. Glucose uptake is the rate-limiting step of glucose usage in skeletal muscles, and is mediated by a glucose transporter GLUT4 [14], [15]. Exercise induces GLUT4 transcription [16] and GLUT4 translocation from intracellular vesicles to sarcolemma, which allows more efficient absorption of glucose from the blood [17], [18]. GLUT4 translocation is induced by phosphorylated AMPK [19]. AMPK also directly phosphorylates PGC-1α, a transcriptional coactivator that contributes to the formation of slow type muscle fibers, mitochondrial biogenesis, and increase in energy expenditure in skeletal muscles [20], [21]. In particular, phosphorylated PGC-1α activates transcription of many important genes, such as cytochrome c (CytC), uncoupling protein 3 (UCP3), and pyruvate dehydrogenase kinase 4 (PDK4) [22]. In mice overexpressing PGC-1α or the transcription factor PPARδ, slow fiber content and endurance are markedly increased, and these mice are resistant to high fat induced obesity [20], [23], [24]. Administration of AICAR and GW1516 (agonists of AMPK and PPARδ, respectively) induce muscle fiber type transition, mitochondrial biogenesis, and increases fatty acid metabolism activity and endurance in mice [7]. Signaling pathways involved in fast-to-slow transition are considered as promising targets for prevention of obesity and treatment of type 2 (insulin-independent) diabetes [20], [23], [24].

To test whether MAF induces fast-to-slow transition, increases aerobic metabolism capacity in skeletal muscle and improves endurance capacity, we administered MAF to C57BL/6 mice with or without exercise training. We assessed their endurance capacity. We used plantaris muscle, which is rich in fast fibers and is highly responsive to exercise training [25], to assess several parameters indicative of fast-to-slow transition phenotype. Our data demonstrate that MAF activates exercise training-induced intracellular signaling pathways that involve AMPK, and improves endurance capacity.

## Materials and Methods

### Approval of Animal Experiments

Animal experiments were carried out in a humane manner after receiving an approval (permit number; 08-002) from the Institutional Animal Experiment Committee of the University of Tsukuba, and in accordance with the Regulations for Animal Experiments at the University, and the Fundamental Guidelines for Proper Conduct of Animal Experiments and Related Activities in Academic Research Institutions under the jurisdiction of the Ministry of Education, Culture, Sports, Science and Technology of Japan.

### Preparation of MAF from Black Tea

MAF was prepared as described previously [4], with modification. Black tea (25 g, obtained from Mitsui Norin Co., Ltd., Japan) was brewed for 1 min in boiling water, and then allowed to stand for 10 min. The brew was filtered through a double layer of filter paper (ADVANTEC No. 2, Advantec Toyo, Tokyo, Japan). The filtrate was extracted with ethyl acetate 10 times to remove ethyl acetate-soluble tea components, such as caffeine, catechins and flavonoids. The ethyl acetate-soluble fraction was removed from the boiling water extract. The modification of the original preparation method was as follows. The polyphenol substances remaining in the water phase were extracted with butanol at pH 2.0. The acidic butanol extract was chromatographed on a Toyopearl HW-40F column (2.4 cm I.D. ×35 cm, Tosoh, Tokyo, Japan) with a linear gradient of 20% to 50% acetone (1,200 ml) and separated into several fractions. MAF existed in a fraction showing high activity in increasing the mitochondrial membrane potential in *T. pyriformis* [4]. MAF was dissolved in distilled water (DW), and used in exercise training experiments.

### Exercise Training and MAF Treatment

We observed that intake of 0.02% MAF caused a decrease in visceral fat in db/db mice, a model for diabetic dyslipidemia (Fig. S4). Because db/db mice drink almost twice as much water as normal mice, we provided 0.04% MAF (dissolved in DW, *ad libitum* drinking) to wild type (C57BL/6J) mice. The dose of MAF administered through water intake was indicated in mg of MAF/g of body weight (Fig. S5). The MAF intake group in this study showed normal body mass, normal water and diet intake, and normal behavior, indicating that MAF intake at this level was safe. To investigate the effects of MAF and exercise, male C57BL/6J mice (6 weeks old) (Charles River Lab., Yokohama, Japan) were divided into four groups (8 animals per group), with average running time in each group almost equal (800 s): (1) non-trained and DW-intake (C), (2) non-trained and 0.04% MAF-intake (CM), (3) exercise-trained and DW-intake (T), and (4) exercise-trained and 0.04% MAF-intake (TM) groups. After an initial running endurance test (week 0), the mice in the T and TM groups were trained on a treadmill at 15 m/min for 30 min for 9 weeks. Each week's training was performed daily for 5 days (slope angle: 0%), then a test was performed on the 6^th^ day, with no training on the 7^th^ day. DW-intake groups drank DW, and MAF-intake groups drank 0.04% MAF. Running endurance tests were performed as follows. After mice were acclimatized to moderate treadmill running, they ran on a treadmill tilted 10% uphill starting at a speed of 10 m/min for 4 min. Every subsequent 4 min, the speed was increased by 2 m/min until mice were exhausted or a maximal speed reached 36 m/min. Exhaustion was defined as the inability of the mouse to remain on the treadmill despite mild electrical stimulus and mechanical prodding. Running time was measured, and running distance was calculated.

### Assay of Plasma Glucose Levels

After the endurance tests of week 9, plasma glucose levels were measured with ONE TOUCH Ultra (Johnson & Johnson, NJ, USA) before and after treadmill exercise at 15 m/min for 30 min (slope angle: 0%).

### Tissue Collection

After assay of plasma glucose levels, all mice were sacrificed and plantaris muscles were collected in 1.5 ml sample tubes, frozen in liquid nitrogen and preserved at −80°C.

### Real-time PCR

Total RNA from plantaris muscle was isolated using SV Total RNA Isolation System (Promega, Madison, WI, USA). Semi-quantitative real-time PCR was performed to examine GLUT4 mRNA levels using SYBR Green PCR master mix (Applied Biosystems, Foster City, CA, USA) on an ABI PRISM 7500 sequence detection system. PCR conditions were as follows: an initial denaturation for 10 min at 95°C, 40 cycles of 15 s at 95°C and 60 s at 60°C, and a dissociation treatment for 15 s at 95°C, for 60 s at 60°C and for 15 s at 95°C. Data were analyzed by relative quantitation using a standard curve and normalized to cyclophilin. The following specific primers were used: GLUT4, forward, 5′-CCGCGGCCTCCTATGAGATACT-3′, and reverse, 5′-AGGCACCCCGAAGATGAGT-3′; PGC-1α: forward, 5′-TGCGTGTGTGTATGTGTGTG-3′, and reverse, 5′-GCCTTGTTCGTTCTGTTC-3′; cyclophilin, forward, 5′-CGATGACGAGCCCTTGG-3′, and reverse, 5′-TCTGCTGTCTTTGGAACTTTGTC-3′.

### Electrophoretic Separation of Skeletal Muscle MHC Isoforms

Plantaris muscles were homogenized in the lysis buffer (50 mM HEPES (pH 7.9), 150 mM NaCl, 1 mM EDTA, 20 mM sodium pyrophosphate, 100 mM NaF, 2 mM Na_3_VO_4_, and 1% Triton X-100). Protein concentrations in the lysates were measured by using a BCA protein assay kit (Thermo Fisher Technology, Waltham, MA, USA). The lysates were solubilized in the SDS sample buffer (62.5 mM Tris-HCl (pH 6.8), 2% sodium dodecyl sulphate, 25% glycerol, 5% 2-mercaptoethanol and 0.01% bromophenol blue), adjusted to equal protein concentrations, and incubated at 37°C for 1 h. The lysates were separated by SDS polyacrylamide gel electrophoresis (SDS-PAGE) using 8.5% running gel and 4.75% stacking gel. The running conditions were 2 h at 100 V and then 22 h at 240 V. The gels were stained with Coomassie Brilliant Blue, dried, and protein bands were quantified with NIH Image v. 1.62 software (National Institutes of Health, Bethesda, MD, USA). Although we could not discriminate between IIa and IId/x, we considered these isoforms were all intermediate forms for fast-to-slow transition.

### Western Blotting

The samples used in MHC analysis were separated by electrophoresis on 10% or 15% polyacrylamide gels and transferred to PVDF membranes (Millipore, Bedford, MA, USA). The membranes were blocked with 2–5% non-fat dry milk (Snow Brand Milk Products, Tokyo, Japan) or Blocking One (Nacalai Tesque, Kyoto, Japan) and probed with the primary antibodies against the following proteins: total and phosphorylated (Thr172) AMPK α subunit (1/1,000 dilution, Cell Signaling Technology, Beverly, MA, USA), PGC-1α (1/500 dilution, Chemicon International, Temecula, CA, USA), PPARδ (1/1,000 dilution, Affinity BioReagents, Golden, CO, USA), cytochrome c (1/1,000 dilution, BD Biosciences, San Jose, CA, USA), COXIV (1/1,000 dilution, Molecular Probes, USA), myoglobin (1/30,000 dilution, Santa Cruz Biotechnology, Santa Cruz, CA, USA), GLUT4 (1/10,000, Chemicon International), β-actin (1/10,000 dilution, Santa Cruz Biotechnology) and anti-β-tubulin (1/10,000 dilution, Sigma, USA). The membranes were then washed with TBS-T (TBS with 0.1% Tween-20) and probed with alkaline phosphatase-conjugated anti-mouse or anti-rabbit IgG secondary antibodies (Biosource International, Camarillo, CA, USA). Protein bands were visualized by using a BCIP-NBT solution kit (Nacalai Tesque, Kyoto, Japan) and quantified with NIH Image v. 1.62 software.

### Statistical Analysis

Between-group differences were determined by Student's t-test or one-way ANOVA followed by Tukey's post hoc test. The statistical software was IBM SPSS Statistic.

## Results and Discussion

### MAF Intake with Endurance Exercise Improves Endurance Capacity

**Figure 1:**
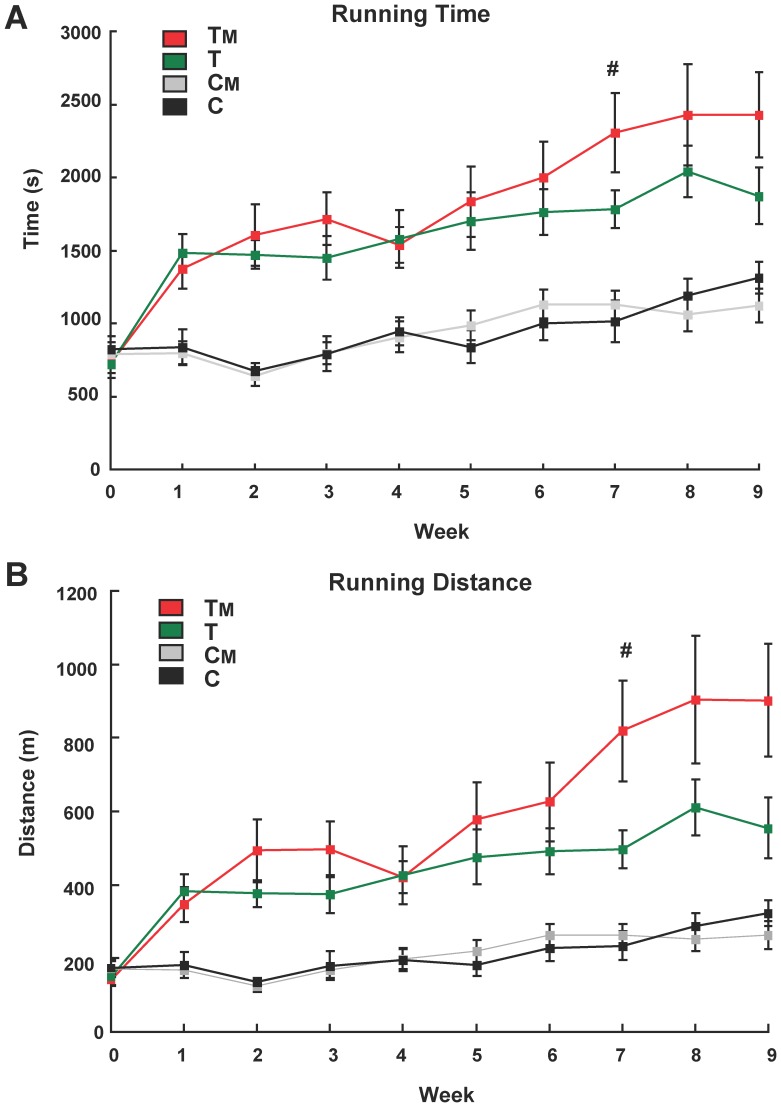
MAF intake improves exercise training-induced running endurance. Effects of MAF on running time (**A**) and distance covered (**B**) until exhaustion were estimated by an endurance measurement test using the treadmill in the C, CM, T, TM groups at weeks 0–9. #, statistically significant difference between the T and TM groups (p<0.05, Student's t-test). Error bars indicate SE (n = 7 or 8).

To examine the effect of MAF, exercise training, or both on endurance capacity in mice, we performed endurance tests weekly for 9 weeks and compared running time and distance among Non-Tr.+DW (C), Non-Tr.+MAF (CM), Tr.+DW (T) and Tr.+MAF (TM) groups (Fig. 1A, B). Increases in running time and distance were slow in the C and CM groups and indistinguishable between those two groups. Therefore, MAF intake has no effect on endurance capacity in the absence of training. Running time and distance in the T and TM groups increased about twofold after 1 week (by approximately 750 s and 200 m, respectively). Despite continued exercise in the T group during weeks 1–9, the differences in the running time and distance between the T group and C or CM groups remained stable (approximately 750 s and 200 m, respectively). In contrast, starting from week 5, the running time and distance increased faster in the TM group than in the other groups (Fig. 1A, B). At week 7, there were significant differences in both the running time and distance between the T and TM groups (Fig. 1A, B). These data demonstrate that MAF intake with endurance exercise significantly improves exercise training-stimulated running endurance. It would be interesting to test whether MAF intake produces similar effects in humans.

### MAF Intake with Endurance Exercise Induces Oxidative Fiber Formation in Skeletal Muscle

**Figure 2:**
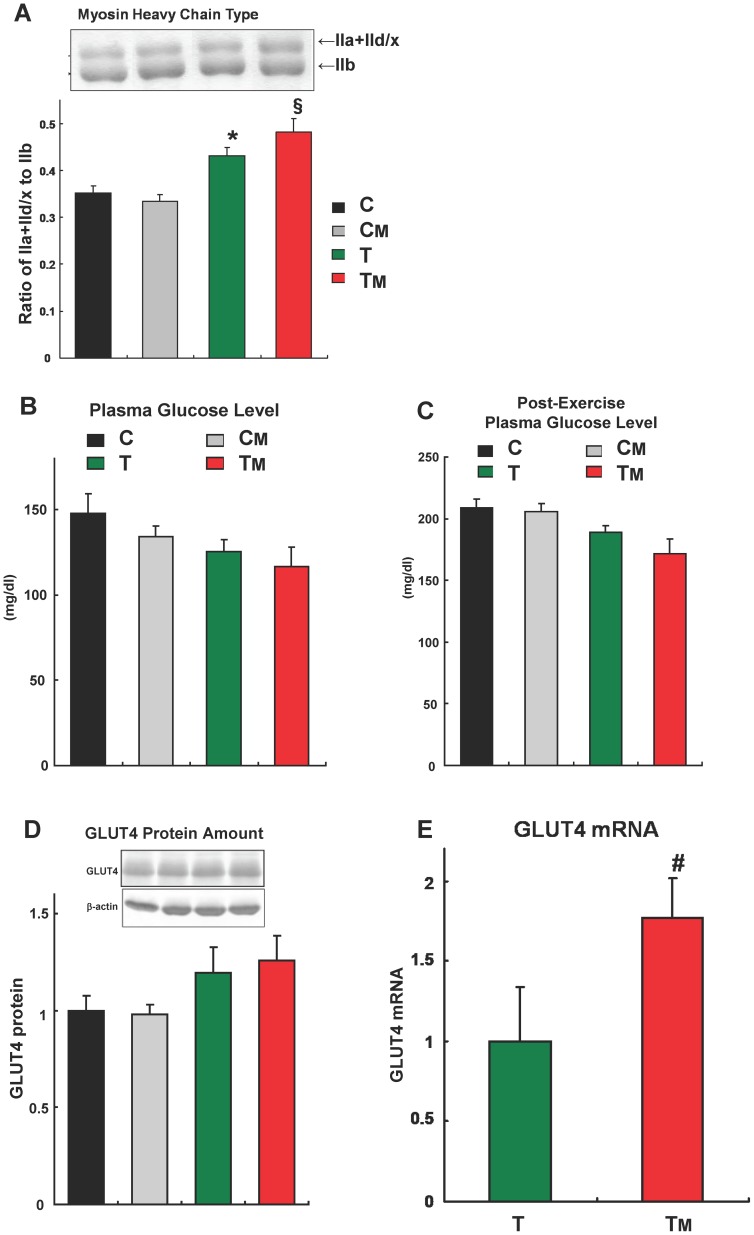
MAF intake increases exercise training-induced oxidative fiber formation in skeletal muscle. **A.** The ratio of myosin heavy chains (IIa+IId/x) to IIb in plantaris muscle was assessed after 9 weeks of endurance training. Inset, a representative Coomassie-stained gel. * and §, statistically significant difference *vs*. C group (§: p<0.01, *: p<0.05, one-way ANOVA followed by Tukey's post hoc test). Error bars, SE (n = 7 or 8). **B, C.** Plasma glucose levels in sedentary state (**B**) and post-exercise (**C**) after 9 weeks of endurance training. Error bars, SE (n = 7 or 8). **D.** GLUT4 protein expression in all 4 groups, measured by Western blotting. Data were normalized to β-actin. Error bars, SE (n = 8). **E.** GLUT4 mRNA expression in the T and TM groups, measured by real-time PCR. #, statistically significant difference (p<0.05, Student's t-test). Data were normalized to cyclophilin. Error bars, SE (n = 8).

Endurance exercise drives the formation of oxidative type fibers and improves endurance capacity [26]. We speculated that MAF intake with exercise training may increase the proportion of slow muscle fibers. We compared the expression levels of oxidative type IIa+IId/x and glycolytic type IIb MHC (Fig. 2A), and found a statistically significant increase in the MHC IIa+IId/x to IIb ratio in both groups that underwent endurance training. MAF intake with exercise training showed a tendency to increase the IIa+IId/x to IIb ratio by 12%, as compared to the T group.

The ratio of type IIa+IId/x fibers to all fibers (type I + IIa + IId/x + IIb) in plantaris increased from 15.3% (sedentary group) to 31.7% (trained group). The ratio of type IIa+IId/x fibers in the trained group reach a plateau during the fast-to-slow transitions, so 32∼33% is considered the physiological maximum ratio of type IIa+IId/x fibers to all fibers in plantaris [25]. In this experiment, the ratio of type IIa+IId/x fibers to all fibers in plantaris in the T group was 30.1%, while that in the TM group was 32.5%, which is really the maximum ratio (Fig. 2A); this result shows that MAF intake enhanced exercise-stimulated fast-to-slow transition degree as far as the limit.

### MAF Intake with Endurance Exercise Stimulates GLUT4 mRNA Expression

After the completion of the 9 week endurance training, we compared plasma glucose levels in the four groups in the sedentary state. The TM group showed a tendency (which was not statistically significant) toward decreased plasma glucose levels as compared with the T group (Fig. 2B). A similar tendency was observed after the mice ran for 30 min (Fig. 2C). We speculate that MAF intake in mice enhances the effect of training on the decrease in plasma glucose levels.

Glucose uptake into skeletal muscle is mediated by GLUT4, and endurance exercise increases its expression [14], [16], [27]. We measured GLUT4 protein level in the plantaris muscle in all groups and GLUT4 mRNA level in the two trained groups. Training slightly (though not significantly) increased GLUT4 protein levels, whereas MAF had no effect (Fig. 2D). In contrast, MAF significantly (by approximately 80%) increased GLUT4 mRNA levels in trained mice (Fig. 2E). We speculate that one possible reason for the absence of significant up-regulation of GLUT4 protein despite strong up-regulation of its mRNA may be that MAF induces GLUT4 protein turnover (i.e. accelerates both its synthesis and degradation).

### MAF Intake with Endurance Exercise Activates AMPK

**Figure 3:**
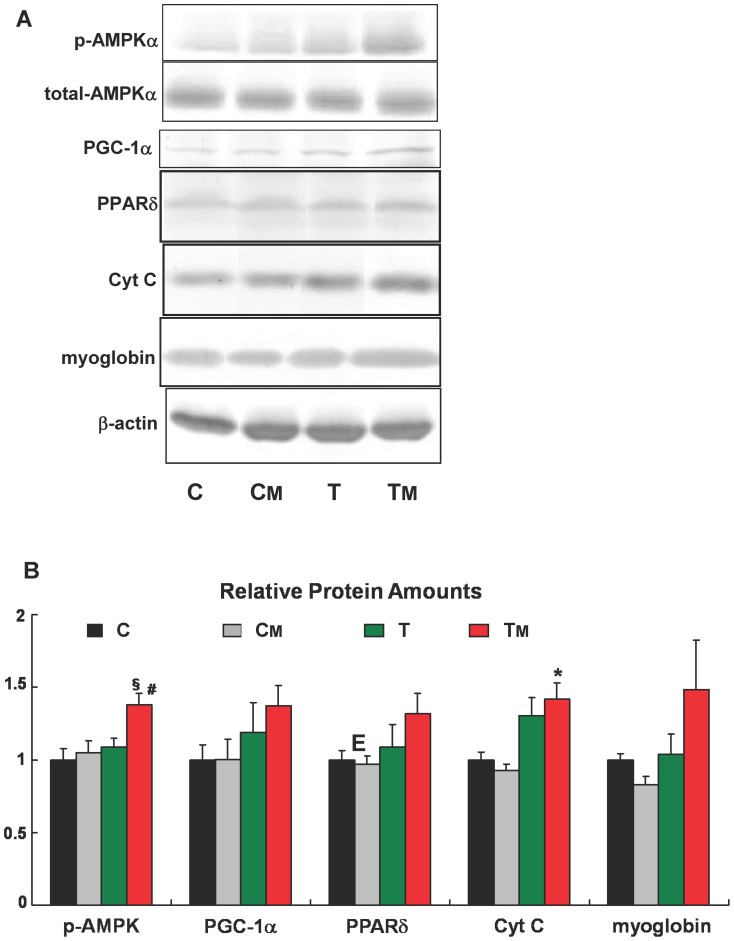
MAF intake increases exercise training-induced factors involved in fast-to-slow transition. AMPKα phosphorylation and expression levels of PGC-1α, PPARδ, cytochrome c, and myoglobin in each group were measured by Western blotting. Representative blots (**A**) and quantification (**B**) are shown for each protein. p-AMPK was determined as p-AMPKα/total AMPKα ratio. Other proteins are normalized to β-actin. *, and §, statistically significant difference between the C and T_M_ groups. #, statistically significant difference between the T and TM groups (* and #: p<0.05, §: p<0.01, one-way ANOVA followed by Tukey's post hoc test). Error bars, SE (n = 8).

We examined the effects of MAF intake on AMPK, PGC-1α, and PPARδ, which are known as critical factors in the fast-to-slow transition in skeletal muscle [20], [23], [24], [28], as well as on cytochrome c and myoglobin. We measured the levels of these proteins and AMPK phosphorylation by Western blotting (Fig. 3). Fig. 3 suggests that there may be some increase in the levels of PGC-1α, PPARδ, and myoglobin in the TM group as compared to those in the T group.

AMPKα phosphorylation was significantly increased (by 27%; p<0.05) in the TM group as compared to the T group (Fig. 3). These data suggest that MAF intake with endurance exercise activates AMPK. Interestingly, p-AMPKα levels in the T group were similar to those in the C and CM groups. In other words, 9 weeks of exercise training did not promote AMPK phosphorylation in the T group. We suggest that this may explain why the increase rates in running time and distance from week 1 to week 9 were similar in the T, C and CM groups. MAF intake promotes AMPK phosphorylation only in the exercise-trained state (i.e. in low ATP state), but not in the sedentary state. Therefore, we speculate that MAF may promote AMPK phosphorylation via AMPKK such as LKB1 and CaMKK, known to phosphorylate AMPK at high AMP/ATP ratios [11], [12]. Therefore, regular exercise may promote the ability of MAF to interfere with receptors upstream of AMPK (such as adiponectin, leptin and/or adrenaline receptors [29]–[31]) or other target proteins. Our data are consistent with a central role of AMPK in orchestrating the synergistic effect of MAF and regular exercise on endurance capacity.

AMPK reportedly affects GLUT4 mRNA levels via PGC-1α expression/phosphorylation [32]. MAF intake with endurance exercise activates AMPK and increases PGC-1α (Fig. 3). Therefore, MAF may regulate the link among AMPK phosphorylation, PGC-1α expression and transcription of GLUT4 mRNA.

### Conclusions

This study demonstrated for the first time that intake of MAF from black tea and exercise training synergistically activates AMPK, increases GLUT4 mRNA expression, promotes fast-to-slow transition in plantaris skeletal muscle and improves endurance, whereas intake of MAF without exercise has no effect. Our work suggests that MAF may amplify exercise-stimulated signaling, but is not sufficient *per se* to initiate such signaling, and warrants further investigation of the molecular mechanisms underlying the effects of MAF.

Metabolism in skeletal muscle, including its aerobic capacity, can be controlled in part by voluntary exercise. Our study suggests that MAF may further enhance this exercise effect and, if the effects in humans are the same as those we have demonstrated in mice, can be considered as a candidate supplement for exercise training to develop physical endurance in athletes.

## Supporting Information

Figure S1 **Concentration-dependent effects of MAF on mitochondrial membrane potential and intracellular ATP level in C2C12 myotubes.** **A.** Computerized quantification of rhodamine123 fluorescence in the C2C12 myotubes after treatment of MAF for 4 h. **B.** Luciferase-based quantification of the cellular ATP level in the C2C12 myotubes after treatment of MAF for 4 h. n = 4. Values represent means ± SE. *P<0.05 vs. control group (0 µg/ml, light bars).
(TIFF)

Figure S2 **Effects of MAF on gene expression of PGC-1α and the protein amounts of PGC-1α and PPARδ in C2C12 myotubes.** **A.** The amount of PGC-1α mRNA after treatment of MAF for 4 h. PGC-1α mRNA level was determined by semi-quantitative real time PCR analysis, and normalized to that of cyclophilin. n = 3. The protein amounts of PGC-1α (**B**) and PPARδ (**C**) after treatment of MAF for 5 h/day during 3 days. The protein amounts were determined by western blots and densitometric quantification. β-tubulin was used as an internal control. n = 4. Values represent means ± SE. *P<0.05 vs. control group (0 µg/ml, light bars).
(TIFF)

Figure S3 **Expression of mitochondrial respiratory proteins after treatment of MAF for 5 h/day during 3 days in C2C12 myotubes.** Representative blots and densitometric quantification of cytochrome c (CytC) (A) and cytochrome c oxidase subunit IV (COXIV) (B). β-tubulin was used as an internal control. n = 4. Values represent means ± SE. *P<0.05 vs. control group (0 µg/ml, light bars).
(TIFF)

Figure S4 **Effects of MAF on visceral fat weights in db/db mice.** Visceral fat weights of *db/db* mice taken in DW (control), MAF or EGCG were measured on the final day of the experiment. Value is expressed as the mean ± SEM. Asterisk shows significant difference from DW (*P*<0.05).
(TIFF)

Figure S5 **Changes of MAF intake and body weights during 9 weeks endurance training.**
(TIFF)

Text S1 Supplemental Text.
(DOC)
