# Supplementary figures and images for: Black Tea High-Molecular-Weight Polyphenol Stimulates Exercise Training-Induced Improvement of Endurance Capacity in Mouse via the Link between AMPK and GLUT4
Source: PLoS One. 2013 Jul 26;8(7):e69480. doi: 10.1371/journal.pone.0069480 (PMC3724851; doi:10.1371/journal.pone.0069480)

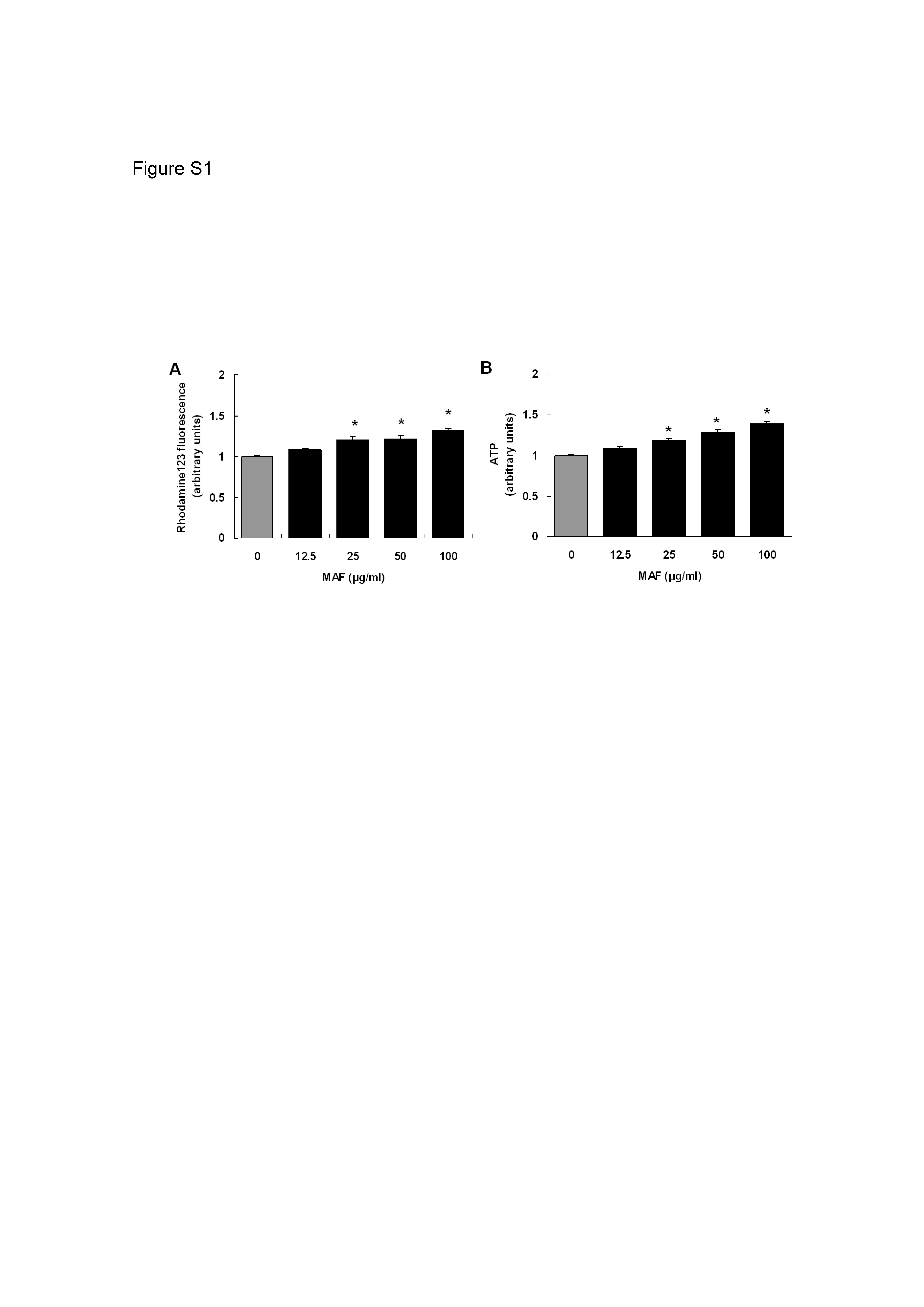

Supplement: Figure S1 — Concentration-dependent effects of MAF on mitochondrial membrane potential and intracellular ATP level in C2C12 myotubes. A. Computerized quantification of rhodamine123 fluorescence in the C2C12 myotubes after treatment of MAF for 4 h. B. Luciferase-based quantification of the cellular ATP level in the C2C12 myotubes after treatment of MAF for 4 h. n = 4. Values represent means ± SE. *P<0.05 vs. control group (0 µg/ml, light bars). (TIFF) [file pone.0069480.s001.tiff]

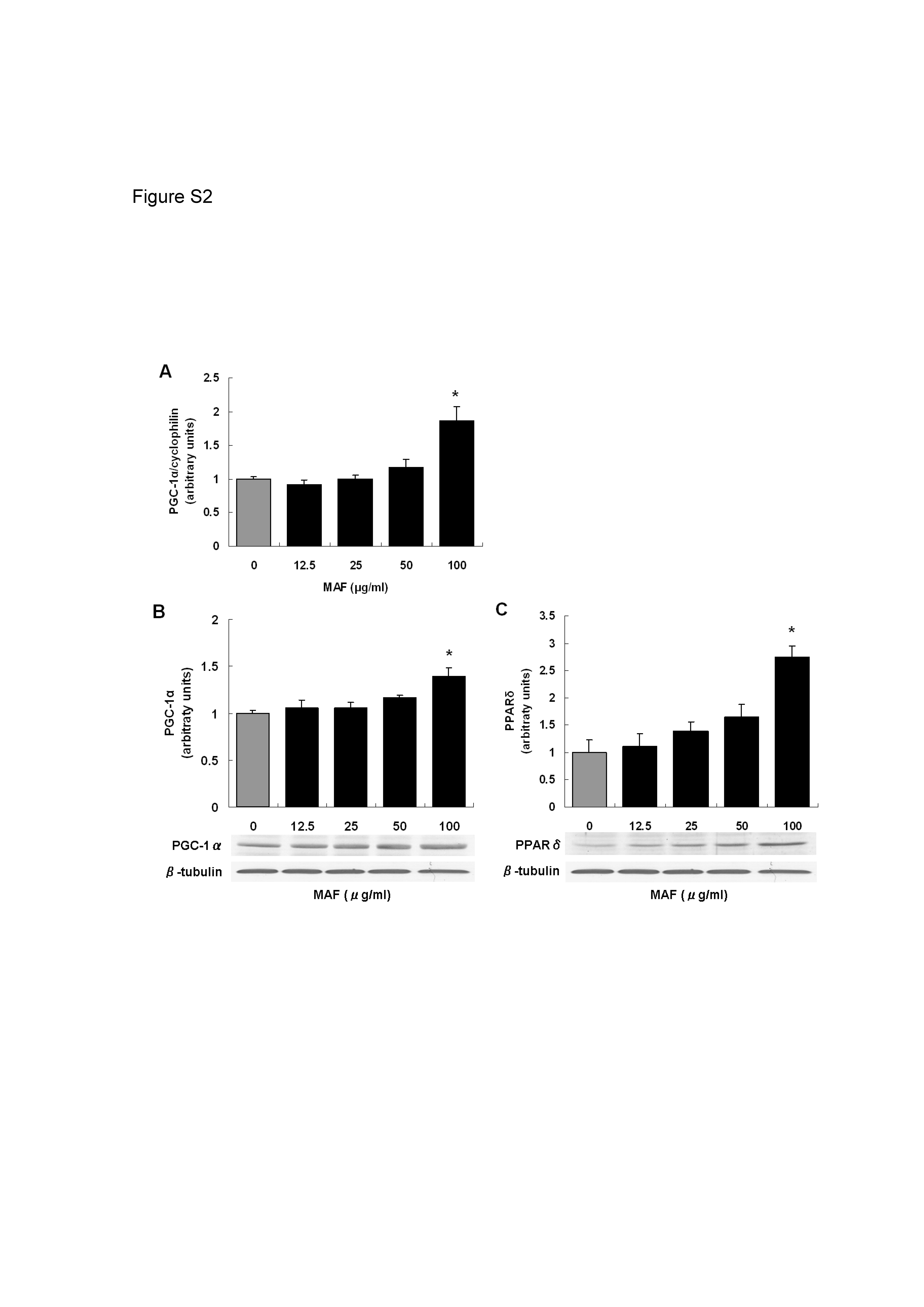

Supplement: Figure S2 — Effects of MAF on gene expression of PGC-1α and the protein amounts of PGC-1α and PPARδ in C2C12 myotubes. A. The amount of PGC-1α mRNA after treatment of MAF for 4 h. PGC-1α mRNA level was determined by semi-quantitative real time PCR analysis, and normalized to that of cyclophilin. n = 3. The protein amounts of PGC-1α (B) and PPARδ (C) after treatment of MAF for 5 h/day during 3 days. The protein amounts were determined by western blots and densitometric quantification. β-tubulin was used as an internal control. n = 4. Values represent means ± SE. *P<0.05 vs. control group (0 µg/ml, light bars). (TIFF) [file pone.0069480.s002.tiff]

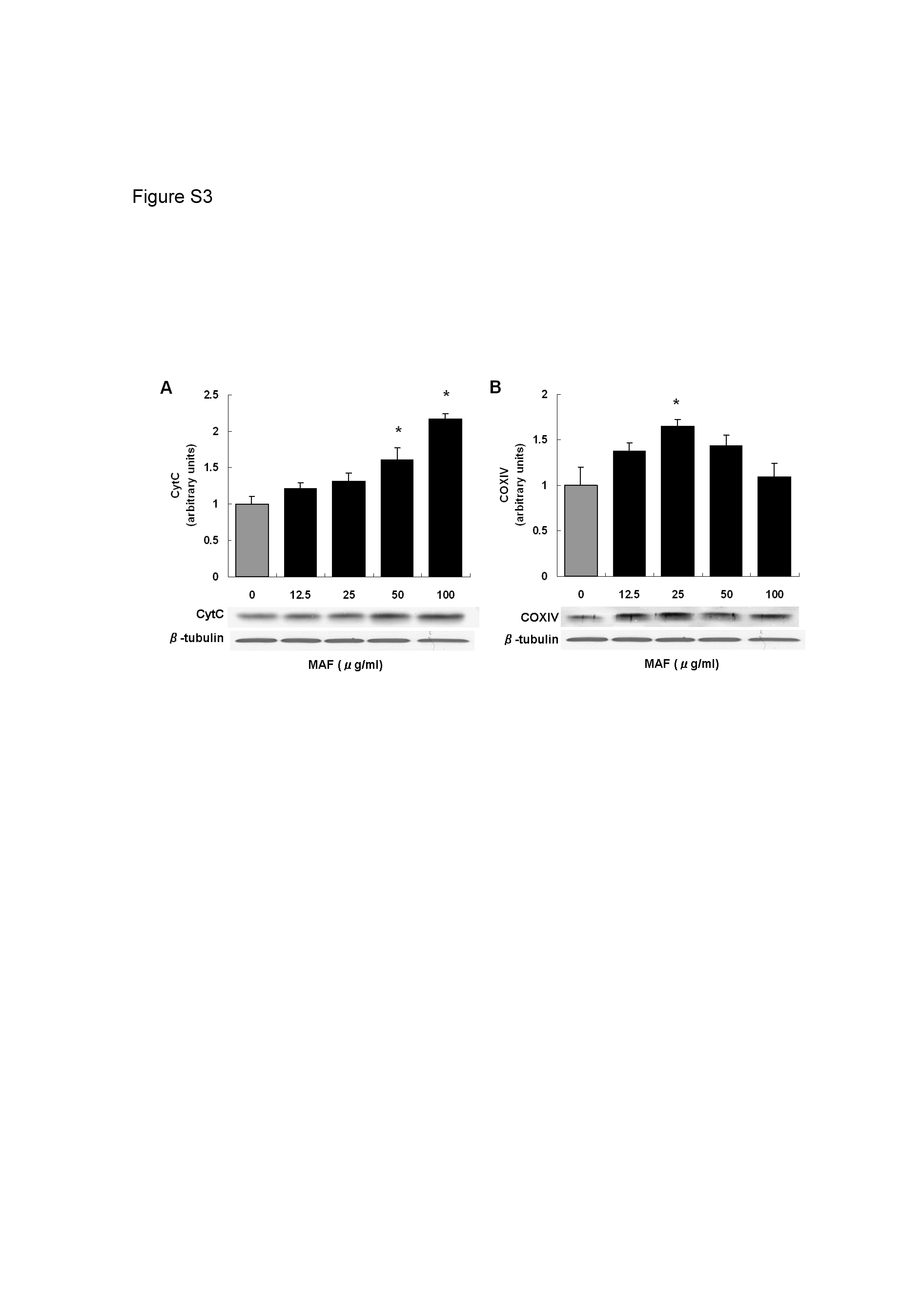

Supplement: Figure S3 — Expression of mitochondrial respiratory proteins after treatment of MAF for 5 h/day during 3 days in C2C12 myotubes. Representative blots and densitometric quantification of cytochrome c (CytC) (A) and cytochrome c oxidase subunit IV (COXIV) (B). β-tubulin was used as an internal control. n = 4. Values represent means ± SE. *P<0.05 vs. control group (0 µg/ml, light bars). (TIFF) [file pone.0069480.s003.tiff]

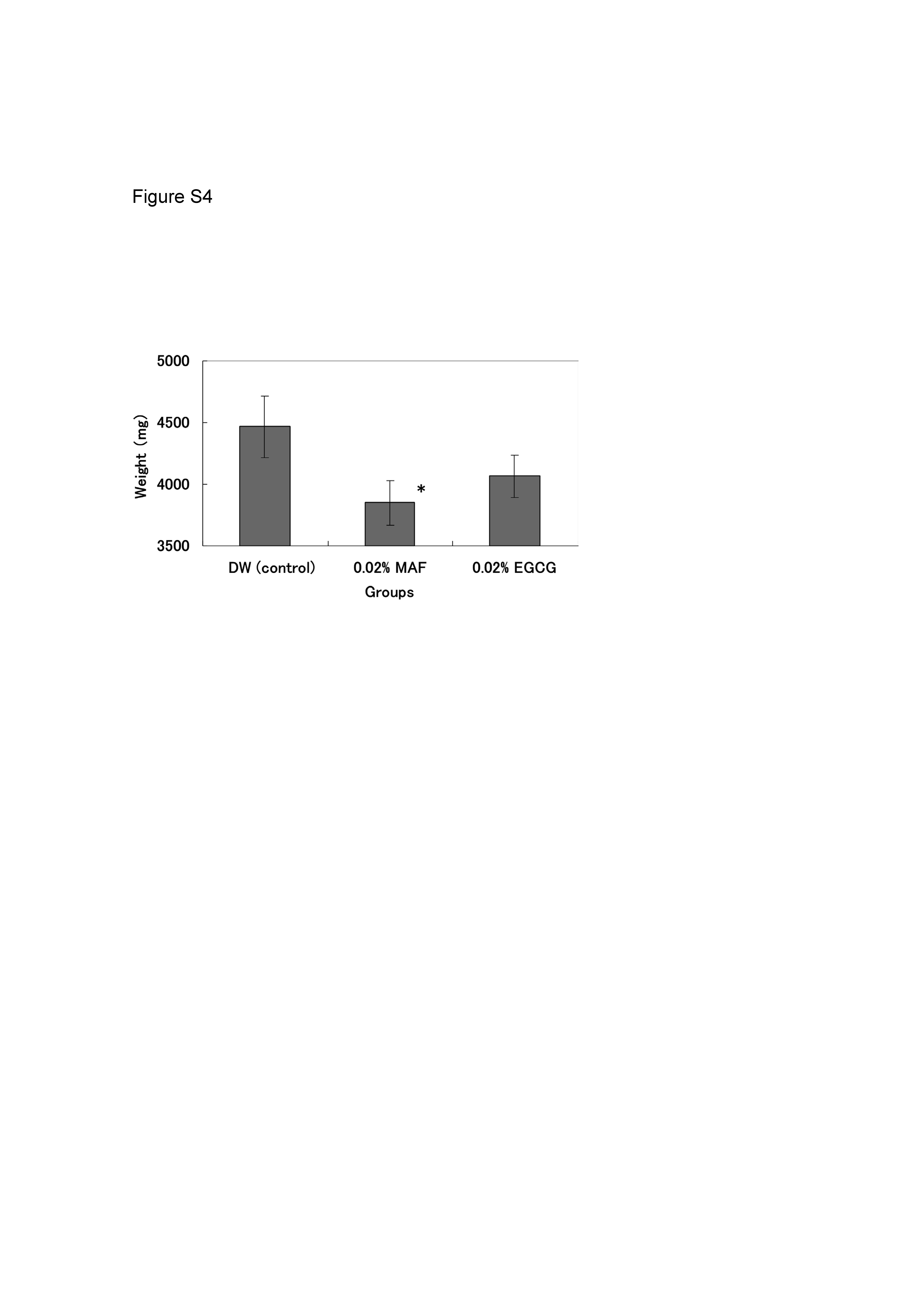

Supplement: Figure S4 — Effects of MAF on visceral fat weights in db/db mice. Visceral fat weights of db/db mice taken in DW (control), MAF or EGCG were measured on the final day of the experiment. Value is expressed as the mean ± SEM. Asterisk shows significant difference from DW (P<0.05). (TIFF) [file pone.0069480.s004.tiff]

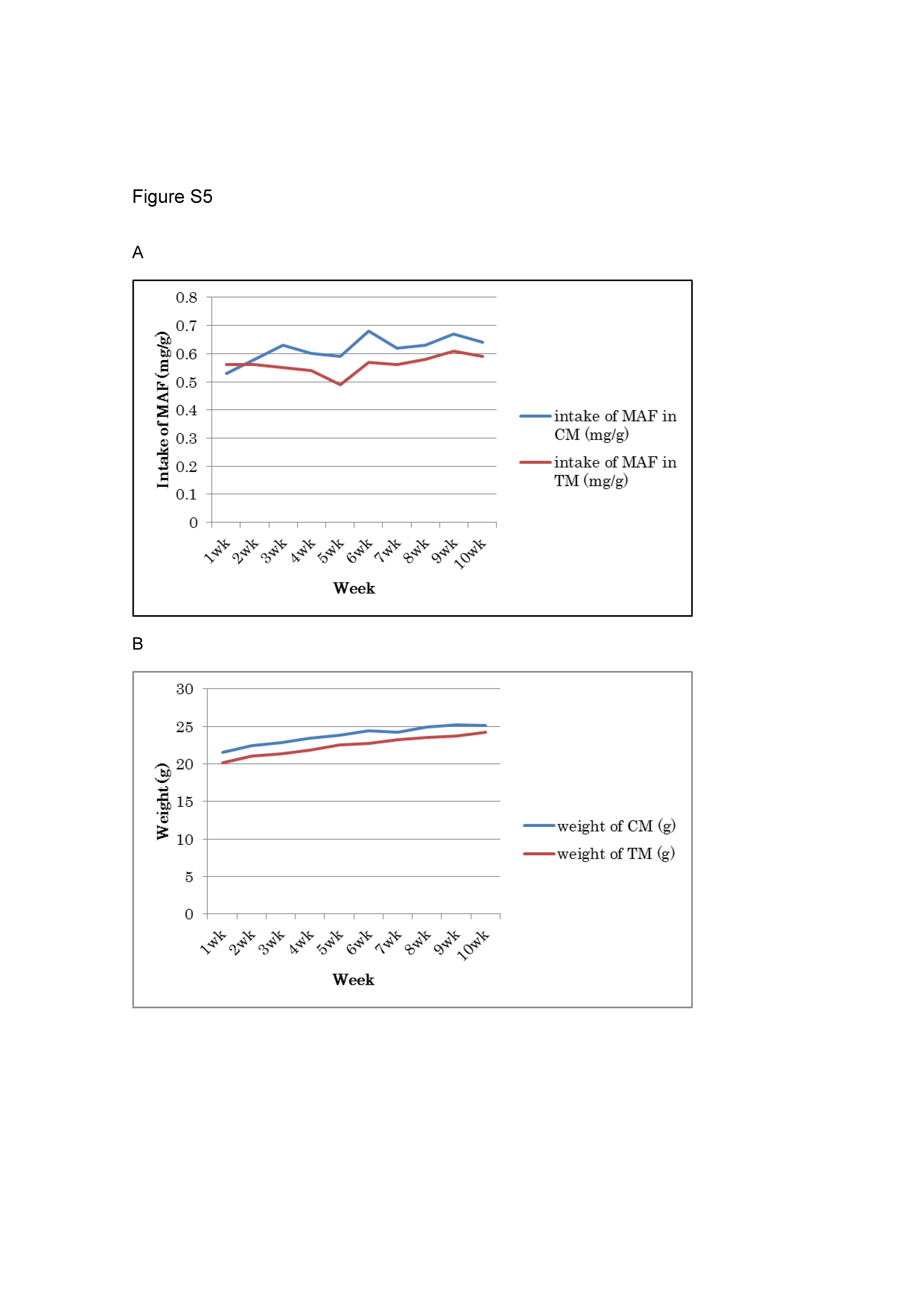

Supplement: Figure S5 — Changes of MAF intake and body weights during 9 weeks endurance training. (TIFF) [file pone.0069480.s005.tiff]
