# Supplementary material for: Black Tea High-Molecular-Weight Polyphenol Stimulates Exercise Training-Induced Improvement of Endurance Capacity in Mouse via the Link between AMPK and GLUT4
Source: PLoS One. 2013 Jul 26;8(7):e69480. doi: 10.1371/journal.pone.0069480 (PMC3724851; doi:10.1371/journal.pone.0069480)
Supplement: Text S1 — Supplemental Text. (DOC) [file pone.0069480.s006.doc]

**Supplemental Text**

**Cell Culture**

Cell cultures were maintained at 37°C in a humidified atmosphere of 5% CO_2_. C2C12 myoblasts were placed on collagen-coated 6- or 96-well plates (TPP, Switzerland) and proliferated in DMEM (Nacalai Tesque, Japan) supplemented with 10% fetal bovine serum (FBS) and an antibiotics mixture (Nacalai Tesque, Japan). When the myoblasts achieved 80% confluence, they were induced to differentiate into myotubes for 7 days by changing the medium to DMEM containing 2% horse serum.

**MAF Treatment**

On the 8th day after differentiation, we pre-exposed the myotubes to serum-free DMEM for 12 h before the following experiments in order to reduce the effects of transfer to fresh medium. The MAF fraction was dissolved in 5% dimethyl sulfoxide (DMSO) and added to DMEM at final concentrations of 12.5, 25, 50, and 100 μg/ml. DMEM containing the same concentration of DMSO was used as control. Then we incubated the myotubes with or without the MAF under the following conditions: (i) 4 h for the rhodamine123 assay, ATP measurement and PGC-1α mRNA assay; (ii) 5 h/day for 3 days for the protein assay. In case (ii), we switched the medium to DMEM containing 2% FBS after MAF treatment.

**Rhodamine 123 Assay**

Mitochondrial membrane potential was determined using rhodamine123 as described previously with minor modifications [4]. The myotubes were treated with 10 μM rhodamine123 for 30 min at 37°C in the dark. They were then washed in PBS, and the fluorescence intensity was measured in a microplate reader (GENios FL, Tekan, Japan) with excitation at 485 nm and emission at 535 nm.

**Measurement of Intracellular ATP Level**

The intracellular ATP level was measured using CellTiter-Glo Reagent (Promega, USA) in accordance with the manufacturer’s protocol.

**Animals and Treatment.**

Five-week old male BSK.Cg-*+Lepr^db^/+Lepr^db^*/Jcl (*db/db*) mice were purchased from CLEA Japan (Tokyo, Japan), as a type 2 diabetes model mouse. The animals were all individually housed in plastic/steel cages and were maintained for one week prior to the start of the experiment. Intakes of food and water and body weight were measured on a weekly basis for the duration of the study. The *db/db* mice were divided into distilled water (DW) group as a control, epigallocatechin gallate (EGCG) group, and MAF group. Each group contained 5~6 mice. EGCG and MAF were dissolved in DW. The final concentrations of EGCG and MAF were adjusted to 0.02% and administered to the mice. At 10 weeks of administration, *db/db* mice from each of the groups were fasted overnight and narcotized by 15 μl/g/mouse Nembutal. Visceral fat excised from the mice were weighed.
